# COVID-19: Time for Post-Exposure Prophylaxis?

**DOI:** 10.3390/ijerph17113997

**Published:** 2020-06-04

**Authors:** Ivan Gentile, Alberto Enrico Maraolo, Prisco Piscitelli, Annamaria Colao

**Affiliations:** 1 Department of Clinical Medicine and Surgery–Section of Infectious Diseases, University of Naples Federico II, Via Pansini 5, 80131 Naples, Italy; albertomaraolo84@alice.it; 2 UNESCO Chair for Health Education and Sustainable Development, University of Naples Federico II, 80131 Naples, Italy; priscofreedom@hotmail.com (P.P.); amcolao58@gmail.com (A.C.); 3 Section of Endocrinology and Metabolic Diseases, Department of Clinical Medicine and Surgery, University of Naples Federico II, Via Pansini 5, 80131 Naples, Italy

**Keywords:** COVID-19, post-exposure, healthcare, prophylaxis

## Abstract

From a healthcare perspective, infection due to the novel coronavirus SARS-CoV-2 (severe acute respiratory syndrome coronavirus 2) and the ensuing syndrome called COVID-19 (coronavirus disease 2019) represents the biggest challenge the world has faced in several decades. Particularly worrisome are the high contagiousness of the virus and the saturation of hospitals’ capacity due to overwhelming caseloads. Non-pharmaceutical interventions such as quarantine and inter-personal distancing are crucial to limiting the spread of the virus in the general population, but more tailored interventions may be needed at an individual level on a case-by-case basis. In this perspective, the most insidious situation is when an individual has contact with a contagious subject without adequate protection. If rapidly recognized afterwards, this occurrence may be promptly addressed through a post-exposure chemoprophylaxis (PEP) with antiviral drugs. This strategy has been implemented for other respiratory viruses (influenza above all) and was successfully used in South Korea among healthcare workers against the Middle East respiratory syndrome (MERS) coronavirus, by providing people who were exposed to high-risk contacts with lopinavir-ritonavir plus ribavirin. Initial experiences with the use of hydroxychloroquine to prevent COVID-19 also seem promising. Post-exposure chemoprophylaxis might help mitigate the spread of SARS-CoV-2 in the current phase of the COVID-19 pandemic.

The World Health Organization (WHO) defined the coronavirus disease 2019 (COVID-19), which is caused by the severe acute respiratory syndrome coronavirus 2 (SARS-CoV-2), as a public health emergency of international concern [1]. The spread of COVID-19 is reaching alarming figures in many countries. As of 2 May, 2020, 3,267,184 cases of COVID-19 have been confirmed worldwide [2]. Overall, more than 229,971 deaths have been reported thus far, with a case fatality rate of about 7% [2]. In such a situation, it is essential to forecast the epidemic trend in order to take adequate healthcare measures. The attack rate (AR) (i.e., the percentage of exposed patients who will eventually become infected) is a metric that may help to estimate this trend. The AR of SARS-CoV2 is still a matter of debate. The WHO reported an AR of about 3%–10% [3] in household contacts of SARS-CoV-2 patients in the Guangdong Province of China, which is similar to that of influenza viruses (about 10%).

In the absence of a specific vaccine, the strategy of epidemic containment lays mainly in the isolation of both cases and contacts and in social distancing of the entire population. Many countries have taken a series of exceptional containment measures, including locking down entire cities. These measures have high social and economic costs but are necessary to avoid the potential collapse of national health systems.

In this context, a post-exposure prophylaxis (PEP) approach may help to reduce the spread of the COVID-19 epidemic. This procedure consists of the administration of drugs to a subject who has been exposed to an infected patient, in the attempt to reduce the risk of becoming infected. The drugs administered are often the same drugs used to treat patients. This strategy is not novel. Indeed, it is well established in the setting of acute viral respiratory infections. For example, oseltamivir and zanamivir, two antivirals used to treat flu symptoms, are administered to exposed subjects to reduce the risk of secondary cases. In a meta-analysis, oseltamivir and zanamivir prevented flu symptoms recurrence in 67%–89% of subjects who underwent PEP [4].

With respect to the coronavirus responsible for the SARS epidemic, no pharmacological prevention strategies were implemented beyond non-specific measures as active symptom monitoring or home quarantine in some countries in 2002–2004.

On the other hand, in the setting of the coronavirus responsible for the Middle East respiratory syndrome (MERS), a study conducted in South Korea evaluated whether PEP was effective in preventing MERS in healthcare workers (HCWs) after unprotected exposure to infected patients [5]. The study enrolled 22 HCWs receiving ribavirin plus lopinavir/ritonavir within 80 h (median 36 h) of unprotected exposure in the previous 14 days. No HCW in the PEP arm contracted MERS versus 6/21 cases in the control (non-PEP) arm (0% vs. 28.6%, *p* = 0.009) [5]. Overall, 21/22 HCWs undergoing PEP experienced adverse effects, but all were mild. Moreover, there were no discontinuations [5], and the risk/benefit ratio was considered reasonable.

Regarding COVID-19, a study from South Korea evaluated the efficacy and tolerability of a PEP strategy using hydroxychloroquine, which was administered to 211 persons (189 patients and 22 HCWs) who were potential contacts of the index patient, a hospital social worker [6]. The drug was administrated at a dose of 400 mg/die for the period of quarantine (14 days) within a median of 58 h after the detection of the index case. The type of exposure was classified as high-risk exposure in nine cases. PEP was completed in 97% of subjects without serious events. The most common symptoms associated with the drug were diarrhea and skin rash. No subject tested positive at the end of quarantine period. However, no definitive conclusions may be drawn from this study without a control arm.

The results of the aforementioned studies are summarized in Table 1.

To date, there is no approved treatment for COVID-19. Several trials are now evaluating the effects of various types of COVID-19 treatment but, to our knowledge, only a few studies have been designed to evaluate a prophylactic approach in COVID-19 [7].

Given the worldwide spread of the COVID-19 epidemic, the lack of treatment and the very promising results of the MERS study [5], it seems reasonable to evaluate whether a PEP approach could reduce the spread of the infection. However, several aspects remain to be established before conducting such a trial, including the drug to administer, the interval between exposure and PEP onset, as well as the dosage and the duration of administration. The drug to use should be one of those currently under study, namely lopinavir/ritonavir, chloroquine, remdesivir, darunavir/ritonavir, ribavirin, arbidol, neuraminidase inhibitors, peptide (EK1), and RNA synthesis inhibitors. Although these drugs were developed for a vast array of treatments, they are now being repurposed to counter COVID-19 based on potential in vitro efficacy [8].

Another issue is the design of the study. A high-quality randomized controlled trial (RCT) would reveal whether the PEP strategy is effective or not in containing COVID-19. A potential example is an ongoing RCT from Spain (ClinicalTrials.gov Identifier: NCT04304053). However, such a design might be prone to distortions, such as contamination and attrition bias; moreover, a large sample size is required, and results cannot be obtained in a timely fashion. As an alternative, in countries with a high burden of cases, a study comparing the attack rate before and after PEP onset in the area under study may provide initial proof of efficacy more rapidly. Potential enrollees would be people who are in contact with COVID-19 patients, individuals already identified by health authorities, or HCWs after unprotected exposure to COVID-19 patients. In ordinary times, such a study should follow proof of in vitro and in vivo activity of the drug and its therapeutic efficacy. In the present exceptional times, any promising approach should be urgently exploited to contain the epidemic.

In conclusion, should a study of PEP demonstrate efficacy in decreasing the circulation of SARS-CoV-2, this approach could be widely implemented in order to help contain the epidemic and reduce the global burden of the disease. 

## Figures and Tables

**Table 1:** Studies on post-exposure prophylaxis (PEP) in coronavirus infections. MERS: Middle East respiratory syndrome; COVID-19: coronavirus disease 2019.

	Author	Year	Design of the Study	Drug/Drugs	Endpoint	Outcome
MERS	Park S.Y.	2019	Retrospective, comparing two cohorts (intervention versus control)	Lopinavir/ritonavir and ribavirin	New MERS cases	No cases among PEP subjects
COVID-19	Lee S.H.	2020	Retrospective, single cohort (no control group)	Hydroxychloroquine	New COVID-19 cases	No cases among PEP subjects
